# Amyotrophic Lateral Sclerosis-like Syndrome after Chikungunya

**DOI:** 10.7759/cureus.5876

**Published:** 2019-10-09

**Authors:** Felipe César Andrade, Vinicius Vergetti, Gabriella Cozza, Maria Clara Falcao, Gustavo Azevedo

**Affiliations:** 1 Medicine, Faculdade Pernambucana De Saude, Recife, BRA; 2 Medicine, Faculdade Pernambuca De Saude, Recife, BRA; 3 Neurosurgery, Hospital Getulio Vargas, Recife, BRA

**Keywords:** chikungunya fever, arbovirus, amyotrophic lateral sclerosis, diagnostic techniques, critically ill

## Abstract

Amyotrophic lateral sclerosis (ALS)-like syndrome refers to a group of conditions whose outcome is similar to that of amyotrophic lateral sclerosis, but with different characteristics in the initial phase and response to therapy. The involvement of an earlier age group, the subacute course, and the stabilization or improvement of the clinical condition during the treatment are most important. There is still no evidence of an association between amyotrophic lateral sclerosis-like syndrome and chikungunya (CHK) infection in the literature. This report was intended to review this syndrome and present a case that occurred after the epidemic of CHK in Pernambuco in 2016. CHK is a fast-onset febrile illness characterized by intense asthenia, arthralgia, myalgia, headache, and skin rash. Reports range from encephalitis, optic neuritis, myeloradiculitis to Guillain-Barré syndrome, generating drastic sequelae such as mental deficiency, blindness, and persistent paralysis. This is the first case report of a possible association of ALS-like syndrome and chikungunya infection. CHK infection may cause ALS-like syndrome. There is a need for further research in this field to develop therapies for neurological complications such as that of CHK.

## Introduction

Amyotrophic lateral sclerosis (ALS) is a progressive motor neuron disease that is considered a neurodegenerative disease [1-3]. It has a multifactorial pathogenesis, and its etiology is not well elucidated.

Approximately 10% of the diagnoses of ALS have to be reviewed due to anomalous progression of the disease [1-2]. Among them, ALS-like syndrome refers to a group of conditions with different characteristics in the initial phase and response to therapy [1-3]. In the definition of the ALS-like syndrome, the involvement of an earlier age group, the subacute course and the stabilization or improvement of the clinical condition during the treatment are the most important [4-6]. 

Case reports of patients who developed ALS-like syndrome indicated that in most cases, their progression was rapid. Loss of strength, initially asymmetrical, was observed, associated with hyperreflexia with extensive plantar responses, and no sensory or sphincter dysfunction were reported [7-8].

Most of the cases had their development associated with human immunodeficiency virus (HIV) infection, showing improvement when antiretroviral therapy (ART) was instituted. The probable cause of the syndrome in these patients was the increased viral replication in the central nervous system (CNS) or cerebrospinal fluid (CSF). ALS-like syndrome was also described following human T lymphotropic virus (HTLV)-1 and human foamy virus (FV) [8-11].

There is still no evidence of an association between ALS-like syndrome and chikungunya (CHK) arbovirus infection in the literature. This report was intended to present a case that occurred after the epidemic of Zika and CHK in Pernambuco, Brazil in 2016 with characteristics of such syndrome, as well as its clinical outcome despite employing therapies.

## Case presentation

A 31-year-old woman presented with pain in lower limbs after seven months of suspected arboviral disease during Zika and CHK epidemics in Pernambuco, Brazil, evolving with gradual asymmetric loss of strength in the lower limbs.

There was hyporeflexia in lower limbs with preservation of superficial and deep sensitivity and muscle tone. She underwent electroneuromyography (ENMG) with axonal motor neuronal involvement in lower limbs without signs of demyelination. CSF serologies were negative (HIV, HTLV, Toxoplasmosis, Tuberculosis, Syphilis, Cryptococcosis, Herpes virus, Cytomegalovirus, Epstein-Barr virus, Varicella-Zoster virus) and cellularity was normal (0.66), but high protein concentration (183 mg/ml). Magnetic resonance images (MRI) of the brain and spinal cord were normal.

The patient underwent pulse therapy with dexamethasone for five days, without improvement in the condition and progression of the motor deficit. She was administered intravenous immunoglobulin (IVIg) 2g/kg for five days, with a slight improvement in the strength of the lower limbs. After two days, a new ENMG revealed intense and active denervation of multiple motor nerves and lumbosacral roots (multiradiculoneuropathy) with electrophysiological integrity of the upper limbs.

Serum serologies for arboviruses revealed only high levels of IgM for CHK, with the rheumatological tests, serologies and protein electrophoresis of CSF, oncological screening and biochemical findings being normal.

She underwent two new infusions of IVIG, evolved with worsening of strength in the lower limb, loss of trunk strength and upper limbs, as well as osteotendinous hyperreflexia in the four limbs. After two months, she presented dyspnea on minimal exertion and dysphonia. Spirometry revealed severe restrictive disorder and daily bilevel positive airway pressure (BiPAP) machine was indicated.

There was a progressive worsening of motor paresis in the lower limbs; aquileus reflexes were abolished, patellar abolished on the right and diminished on the left; paresis of upper limbs; biceps and tricipital hyperreflexia on the left; superficial and deep sensitivity preserved. She also had a loss of strength in the trunk and bilateral Hoffman sign.

She presented respiratory insufficiency, even after new pulse therapy with methylprednisolone for five days, being intubated, requiring continuous mechanical ventilatory assistance. The disease course was thus clinically progressive and worsening, and five plasmapheresis sessions were performed, without clinical improvement. New ENMG revealed a pattern consistent with diffuse axonal motor neuropathy with no fasciculations and no conduction block, without bulbar involvement, without dysphagia. She presented joint pain with joint edema and hypotrophy, flaccidity, osteotendinous hyperreflexia. In this phase, polymerase chain reaction (PCR) for arboviruses was negative in CSF and synovial fluid of the knees, but IgG for CHK was elevated in the CSF. Serology for Zika virus was negative. She presented signs of arthritis in the hands, in agreement with arbovirus, and was treated with chloroquine. After 188 days of hospitalization in the intensive care unit (ICU), the patient suffered respiratory complications, leading to death.

## Discussion

Similar to ALS, ALS-like syndrome constitutes a diagnosis of exclusion, after other conditions with similar manifestations are discarded. These include inflammatory demyelinating polyneuropathy, multifocal neuropathy with conduction blockages, motor polyradiculopathy, multiple organic mitochondrial dysfunction syndrome, multifocal motor neuropathy, and cervical spondylosis with myeloradiculopathy [7].

The case report in this article refers to an ALS-like syndrome after an infection by the Chikungunya virus (CHIKV). CHIKV is an arbovirus belonging to the family Togaviridae, genus Alphavirus, transmitted by mosquitoes of the genus *Aedes*, particularly *Aedes aegypti* and *Aedes albopictus*, two invasive and cosmopolitan species. According to the Brazilian Ministry of Health, symptomatic cases can manifest in two forms: typical and atypical [12].

CHK is a fast-onset febrile illness characterized by intense asthenia, arthralgia, myalgia, headache and skin rash. In contrast to other arboviruses, such as dengue, most infected are symptomatic, less than 15% are asymptomatic. Nonspecific signs and less common symptoms include lymphadenopathy, pruritus, and digestive disorders. Severe cases can manifest with encephalopathy and encephalitis, myocarditis, hepatitis and multiple organ failure, these rare forms can be fatal. Its association with neurological sequelae has been increasing [12-13].

Neurological complications have been the main cause of admission in the ICU followed by death. Reports range from encephalitis, optic neuritis, myeloradiculitis to Guillain-Barré syndrome, generating drastic sequelae such as mental deficiency, blindness and persistent paralysis [12-13].

Manifestations directly related to infection appear to occur particularly in children and elderly patients, whereas autoimmune forms should be considered in middle-aged, previously healthy patients, especially after an asymptomatic interval [12-13].

## Conclusions

CHK infection may cause ALS-like syndrome. Further research in this area is essential for developing therapies for neurological complications such as those associated with CHK.

This report points to the difficulty of treating these cases, wherein repeated use of immunotherapeutics with human endovenous Ig, corticosteroids, cyclophosphamide and plasmapheresis sessions were not successful.

## References

[1] Cortés-Vicente E, Pradas J, Marin-Iahoz J (2017). Early diagnosis of amyotrophic lateral sclerosis mimic syndromes: pros and cons of current clinical diagnostic criteria. Amyotroph Lateral Scler Frontotemporal Degener.

[2] Ghasemi M (2016). Amyotrophic lateral sclerosis mimic syndromes. Iran J Neurol.

[3] Bowen LN, Tyagi R, Li W (2016). HIV-associated motor neuron disease: HERV-K activation and response to antiretroviral therapy. Neurology.

[4] Alfahad T, Nath A (2013). Retroviruses and amyotrophic lateral sclerosis. Antiviral Res.

[5] Anand KS, Wadhwa A, Garg J, Mahajan RK (2014). Amyotrophic lateral sclerosis-like presentation in a HIV-positive patient. J Int Assoc Provid AIDS Care.

[6] Moulignier A, Moulonguet A, Pialoux G, Rozenbaum W (2001). Reversible ALS-like disorder in HIV infection. Neurology.

[7] Sinha S, Mathews T, Arunodaya GR (2004). HIV-1 clade-C-associated “ALS”-like disorder: first report from India. J Neurol Sci.

[8] MacGowan DJL, Scelsa SN, Waldron M (2001). An ALS-like syndrome with new HIV infection and complete response to antiretroviral therapy. Neurology.

[9] Verma A, Berger JR (2006). ALS syndrome in patients with HIV-1 infection. J Neurol Sci.

[10] Mahto SK, Gupta PK, Sin gh A, Meena RC (2018). Atypical neurological manifestations of chikungunya fever: two case reports. Indian J Crit Care Med.

[11] Petersen LR, Powers AM (2016). Chikungunya: epidemiology. F1000Res.

[12] Weaver SC (2014). Arrival of Chikungunya virus in the new world: prospects for spread and impact on public health. PLoS Negl Trop Dis.

[13] Nunes MRT, Faria NR, de Vasconcelos JM (2015). Emergence and potential for spread of Chikungunya virus in Brazil. BMC Med.

